# Duhuo Jisheng Decoction ameliorates experimental intervertebral disc degeneration by modulating immune-inflammatory responses through regulation of macrophage polarization

**DOI:** 10.3389/fimmu.2026.1774140

**Published:** 2026-04-21

**Authors:** Yongliang Mei, Liquan Wang, Chao Song, Rui Lv, Xianping Xie, Zongchao Liu

**Affiliations:** 1 Department of Orthopedics, The Affiliated Traditional Chinese Medicine Hospital, Southwest Medical University, Luzhou, Sichuan, China; 2 Southwest Medical University, Luzhou, Sichuan, China; 3 Luzhou Longmatan District People’s Hospital, Luzhou, Sichuan, China

**Keywords:** bioinformatics, Duhuo Jisheng decoction, intervertebral disc degeneration, macrophages, single-cell sequencing technology

## Abstract

**Background:**

The predominant cause of lower back pain (LBP) is intervertebral disc degeneration (IVDD), which has a substantial financial cost to both individuals and society. In the pathophysiology of IVDD, the immune-inflammatory response is crucial. The number of macrophages in the nucleus pulposus (NP) was positively correlated with the degree of IVDD. The extracellular matrix (ECM) disintegration, pyroptosis, and apoptosis of nucleus pulposus cells (NPCs) have all been demonstrated to have a significant impact on IVDD and are closely linked to immune-inflammatory regulation. Meanwhile, we have shown that Duhuo Jisheng Decoction (DHJSD), which modulates the aforementioned pathways, can significantly alleviate IVDD. It’s unclear, nevertheless, if DHJSD can lessen IVDD by regulating macrophage modulation of the immune-inflammatory response.

**Methods:**

In this study, we analyzed the relationship between different subtypes of macrophages and IVDD by using single-cell sequencing. Afterwards, bioinformatics techniques were employed to investigate the primary target genes in IVDD. *In vitro* and *in vivo* experiments were performed to investigate the mechanism of action of DHJSD in ameliorating IVDD by regulating macrophages.

**Results:**

The results of *in vivo* experiments showed that DHJSD was able to modulate macrophages to regulate the immune-inflammatory response to ameliorate IVDD, and the results of *in vitro* experiments showed that DHJSD was able to modulate macrophage polarization to regulate the immune-inflammatory response.

**Conclusion:**

We discovered that IVDD may be lessened by DHJSD by boosting M2 macrophage polarization and suppressing M1 macrophage polarization. IVDD responded better to DHJSD treatment in the intermediate and advanced stages of the disease.

## 1 Introduction

In orthopedics, IVDD is a prevalent persistent condition, and LBP is a heavy health problem and a huge financial burden on people (1). The disc degeneration cannot be substantially reversed by current treatments; they can only provide symptomatic alleviation. This is mainly because the main pathogenic mechanism is not the focus of current IVDD research. A number of investigations have shown that immunological factors, autophagy, cellular senescence, genetic factors, and different cell death processes are intimately associated with IVDD. It is also easy to determine that the immune-inflammatory response is related to all of the aforementioned components (2–6). It is obvious that the immune-inflammatory response is the most important feature in the research of the specific mechanisms of IVDD (6, 7). According to recent research, inflammatory cytokines such as tumor necrosis factor-alpha (TNF-α) and interleukin-1 beta (IL-1β) are typically raised during the development of IVDD, and this leads to an imbalance in ECM creation and breakdown (8, 9). The quantity of macrophages is directly connected with the degree of disc degeneration. Macrophages are important cells in the immune-inflammatory response and are the only inflammatory cells that can enter the closed NP (10–12). At the same time, macrophage phenotypes in the local immune microenvironment of the disc greatly influence ECM metabolic homeostasis, and modulation of macrophage repair of the ECM is expected to ameliorate IVDD (13). It is critical to investigate the involvement of macrophages in the immune-inflammatory response in IVDD in order to more effectively treat it and to better understand its probable mechanism.

We used single-cell sequencing to explore the role of immune cells in IVDD and discovered that macrophages are implicated in its development. We further examined the IVDD samples and discovered that the amount of M1 macrophages rose while the amount of M2 macrophages dropped compared to the black control group. As a result, we feel that examining alterations in macrophage subtypes in IVDD is critical for understanding the underlying pathophysiology and treating IVDD.

The GSE15227, GSE34095, GSE124272, and GSE150408 datasets from the Gene Expression Omnibus database were examined, and we used the databases of GeneCards and the Comparative Toxicogenomics Database (CTD) to collect essential genes for IVDD disease in order to further identify the genes governing the condition. 10 important genes were extracted from the 131 IVDD critical genes that we had discovered, and the differences were statistically examined (14). In the intervertebral disc, aggrecan, together with collagen, is responsible for tissue structure and mechanical function, and it has been clearly demonstrated that aggrecan is essential for determining and maintaining appropriate rigidity of the intervertebral disc tissues (15). Apart from this, matrix metalloproteinase-2 (MMP2), a gene for the ECM-degrading enzyme, is also strongly associated with IVDD (16). Meanwhile, some studies have proved that MMP2 is a key hub gene in IVDD (17, 18). Some studies have also identified TNF-α and IL-1β as key mediators in IVDD and LBP (8, 19). Interleukin-5 (IL-5), also an inflammatory factor, was also found to be highly expressed in IVDD samples (20, 21). Ultimately, we selected aggrecan, collagen 2, MMP2, matrix metalloproteinase-13 (MMP13), IL-5, IL-1β, and TNF-α for further study.

In traditional Chinese medicine, IVDD is typically categorized as “paralysis” or “lumbar paralysis.” Modern pharmacological research along with pertinent clinical investigations has also shown the anti-inflammatory, immunomodulatory, and IVDD-improving effectiveness of DHJSD, a traditional formula for treating IVDD (22–25). Furthermore, by controlling the stromal cell-derived factor 1 (SDF-1)/C-X-C chemokine receptor type 4 (CXCR4)-nuclear factor kappa-light-chain-enhancer of activated B cells (NF-κB) pathway, DHJSD has been shown in earlier research to be effective in treating IVDD by preventing NPCs pyroptosis and, consequently, the inflammatory manifestation of IVDD (26, 27). However, its ability to mediate the immune-inflammatory response by targeting macrophages for the treatment of IVDD has not been validated.

Additionally, we will build an IVDD model in Sprague-Dawley (SD) rats for *in vivo* experiments to examine the mechanism of operation of DHJSD in controlling macrophage polarization to modulate the immune-inflammatory response and thereby ameliorate IVDD. Finally, we will conduct mass spectrometry analysis of DHJSD to investigate the active ingredients of DHJSD. We will conduct further *in vitro* studies to verify whether DHJSD modulates macrophage polarization to modulate the immune-inflammatory response to ameliorate IVDD. In this study, we intend to clarify the regulation mechanism of the immune-inflammatory response of DHJSD in IVDD and further explore the optimal time node of the therapeutic effect of DHJSD in the process of IVDD, which, we believe, will provide value for clinical improvement of patients’ LBP and delay the progression of IVDD and offer a better theoretical foundation for the treatment of IVDD by traditional Chinese medicine.

## 2 Methods and materials

### 2.1 Single-cell data processing and analysis

Cells having a gene count of more than 200 and fewer than 8000, a mitochondrial gene percentage of less than 20%, an erythrocyte gene percentage of less than 3%, and genes expressed in at least three cells were kept out of the dataset during quality control. The Seurat software (version 4.4.0) was used to integrate, cluster, downscale, and display the dataset. The filtered data were first normalized using the NormalizeData function with log normalization, and the top 2,000 highly variable genes (HVGs) were identified using the FindVariableFeatures function. Subsequently, the ScaleData function was applied for scaling, during which the percentages of mitochondrial and hemoglobin genes were regressed out to minimize technical noise in downstream analyses.

Principal component analysis (PCA) was then performed using the RunPCA function, with a total of 50 principal components (PCs) computed. Based on the elbow plot, the top 20 statistically significant PCs were selected for downstream analysis. To correct for batch effects across samples, the Harmony algorithm was applied to the PCA results, using the sample origin (orig.ident) as the grouping variable. The resulting Harmony embeddings were used for constructing cell neighbor graphs, clustering, and UMAP visualization.

After identifying cell clusters, differentially expressed genes (DEGs) for each cluster were identified using the FindAllMarkers function with the Wilcoxon rank-sum test (only.pos = TRUE). DEGs were selected based on an average log2 fold change (avg_log2FC) > 0.75 and an adjusted p-value (p_val_adj) < 0.05. Cell clusters were annotated by integrating the identified marker genes with canonical cell-type-specific marker genes.

Differential expression analysis was performed to assess gene expression differences between groups, and genes were ranked based on their log2 fold change values to generate ordered gene lists. These lists were then mapped to predefined gene sets, and enrichment scores were calculated using the clusterProfiler package, with statistical significance assessed via permutation testing. This approach enabled the identification of significantly enriched pathways, including Kyoto Encyclopedia of Genes and Genomes (KEGG) pathways.

Gene set scores were calculated using the UCell package to evaluate the enrichment level of specific gene sets in individual cells, and the resulting scores were visualized using ggplot.

### 2.2 Bioinformatics data

#### 2.2.1 Microarray data sources and differential gene analysis

The NCBI Gene Expression Omnibus Public Database (GEO) was the source of the gene microarray data for IVDD. There are 31 IVDD samples and 40 normal samples in the GSE15227, GSE34095, GSE124272, and GSE150408 data. Sangerbox 3.0 was used to convert the IVDD data to their corresponding gene names; the “limma” tool was chosen to carry out gene differential analysis and look into how different gene expression levels affect IVDD. The keyword “intervertebral disc degeneration” was searched in the GeneCards and CTD databases. Afterwards, the significant genes for IVDD were obtained by intersecting all the gathered data with the sum of the differential genes of GSE15227, GSE34095, GSE124272, and GSE150408. IVDD-significant genes were analyzed using a protein-protein interaction network (PPI network) restricted to the STRING database of Homo sapiens (https://cn.string-db.org/) with a confidence level greater than 0.4. At the end, heat map analysis of the differential genes was carried out using the “limma” tool of Sangerbox 3.0.

#### 2.2.2 Weighted gene co-expression network analysis

The gene co-expression networks of GSE15227, GSE34095, GSE124272, and GSE150408 were created using the weighted gene co-expression network analysis (WGCNA) program in Sangerbox 3.0 (R software). Based on the clustering tree, the top 50% of genes with the least median absolute deviation were eliminated. After calculating the correlation coefficients between gene pairs, a similarity matrix was created. In order to guarantee the creation of a network that is free of scale, an appropriate soft threshold was used to transform the similarity matrix into an adjacency matrix. A topological overlap matrix was then constructed to calculate the mean network connectivity of every gene. Using the dynamic tree-cutting method, genes with comparable expression profiles were classified into different modules according to the relevant parameters of the block-dimensional module function. Genes in gray modules indicate genes that cannot be allocated to any module. Each module is represented by a different color. Key modules for additional examination were determined by looking at the modules with the greatest absolute values of correlation coefficients. The correlation coefficient between a gene’s expression value and the module’s module identity gene (ME) is known as module affiliation (MM), while the correlation coefficient between a gene’s expression value and its phenotype is known as gene significance (GS). To extract the centered genes, the MM, GS, and weight thresholds were adjusted to 0.7, 0.1, and 0.1, respectively.

#### 2.2.3 Hub gene screening

We obtain the IVDD gene dataset and the major module gene dataset by screening the hub module genes in WGCNA and the significant genes in IVDD. By taking the crossover of these two datasets, we can gain some more plausible IVDD pivotal genes. To acquire the 10 important genes, we filtered the retrieved key genes using the CytoHubba method of the Cytoscape 3.9.1 program. Using a statistical analysis, we extracted the expression of the 10 essential genes once more in order to determine the differences between IVDD and normal people.

### 2.3 *In vivo* experiment

#### 2.3.1 DHJSD pellet preparation

The origin, medicinal composites, and processing technology of DHJSD were standardized based on marker compounds to achieve quality control according to the Chinese Pharmacopoeia 2015 (Chinese Pharmacopeia Commission: Pharmacopoeia of the People’s Republic of China. Chinese Medical Science and Technology Press; Beijing, China, 2010). We obtained DHJSD herbs from Beijing Tongrentang Co. DHJSD formulation: Angelica pubescens f. biserrata, Taxillus chinensis, Eucommia ulmoides, Achyranthes bidentata, Asarum sieboldii, Gentiana macrophylla, Poria cocos, Cinnamomum cassia, Saposhnikovia divaricata, Ligusticum chuanxiong, Panax ginseng, Glycyrrhiza uralensis, Angelica sinensis, Paeonia lactiflora, and Rehmannia glutinosa. According to the composition of this prescription, take 9 grams of Angelica pubescens f. biserrata and 6 grams of the remaining medicinal materials. We have detailed the Chinese name, scientific name, genus, family, medicinal part, and batch number of the above-mentioned medicinal materials in **Supplementary Table 1**. The above-mentioned medicinal materials were centrifuged to filter out impurities. After processing, extraction, concentration, drying, and granulation, DHJSD concentrated granules were finally prepared (this process was completed by Beijing Tongrentang Co., LTD.). DHJSD concentrated pellet preparation powder (5 grams) was added 8 times the amount of 100% ethanol, ultrasonic extraction for 0.5 hour (power 250 W, frequency 40 kHz). It was placed, cooled to room temperature, centrifuged in a centrifuge for 10 minutes (at 12,000 r/min), and the supernatant A was collected. After that, the precipitate was extracted by ultrasonication with 8 times the amount of water for 1 hour (power 250 W, frequency 40 kHz), left to cool to room temperature, centrifuged for 10 minutes in a centrifuge (speed 12000 r/min), and the supernatant B was collected and set aside.

#### 2.3.2 Mass spectrometry

The chemical composition of DHJSD was identified using a Q Exactive Mass Spectrometer, and the collected data were analyzed with the herbal ingredient database, the chemical compositions consulted, and the controls to determine the active substance components in DHJSD.

#### 2.3.3 Construction of IVDD model, DHJSD gavage, and animal grouping

We selected a total of 70 SD male rats for this study. Surgery was performed on 60 3-month-old SD male rats weighing about 300 g. Rats were anesthetized by intraperitoneal injection of 1% pentobarbital at a dose of 40 mg per kg. After successful anesthesia, the caudal discs Co6/7 and Co8/9, which had been identified and localized by X-rays in the previous experiment, were marked. Disinfect the markings with iodophor and alcohol, respectively. And the fibrous annulus was punctured to a depth of 5 mm (puncturing the disc without penetrating the contralateral skin) using a 16-gauge sterile needle, and the needle was rotated *in situ* by 360 degrees after remaining at that depth for 30 seconds. After removing the needle, the hemostasis was halted by applying pressure with a cotton ball. The remaining 10 SD male rats underwent sham surgery (only puncturing the skin without needling the annulus fibrosus) as controls. After the procedure, the rats were rewarmed by placing them next to the heater, and once they awoke from anesthesia, they were put back in their cages.

Our previous research experiments showed that the establishment of the IVDD model could be completed 30 days after the needling modeling (28). To study the curative effect of DHJSD on mild, moderate, and severe IVDD and the optimal time point for treatment, we divided the rats in the abnormal group into three groups according to the time after the needling, i.e., the group at 15 days after the needling (the mild degeneration group), the group at 30 days after the needling (the moderate degeneration group), and the group at 45 days after the needling (the severe degeneration group). Then half of the rats in each of the above three groups were randomly picked as the treatment group and the other half as the model group. The treatment group was gavaged with 6.3 times the equivalent human dose of DHJSD pellets (0.32 g/100 g) once a day in the early morning for 1 month; the model group was gavaged with saline corresponding to body weight. Based on the drug conversion coefficients for humans and rats and the previous experiments on the drug concentration of DHJSD (28), we finally determined the drug concentration and duration. According to the time after the needling and the different interventions (DHJSD and saline), we obtained the following groups and quantities of rats: ten black control group rats, ten rats in the model group 15 days after the needling, ten rats in the model group 30 days after the needling, ten rats in the model group 45 days after the needling, ten rats in the treatment group 15 days after the needling, ten rats in the treatment group 30 days after the needling, and ten rats in the treatment group 45 days after the needling. For experimental convenience, we abbreviated the above groupings as NC, M-15D, M-30D, M-45D, T-15D, T-30D, and T-45D, respectively.

#### 2.3.4 Imaging and histological evaluation

**Figure 1:**
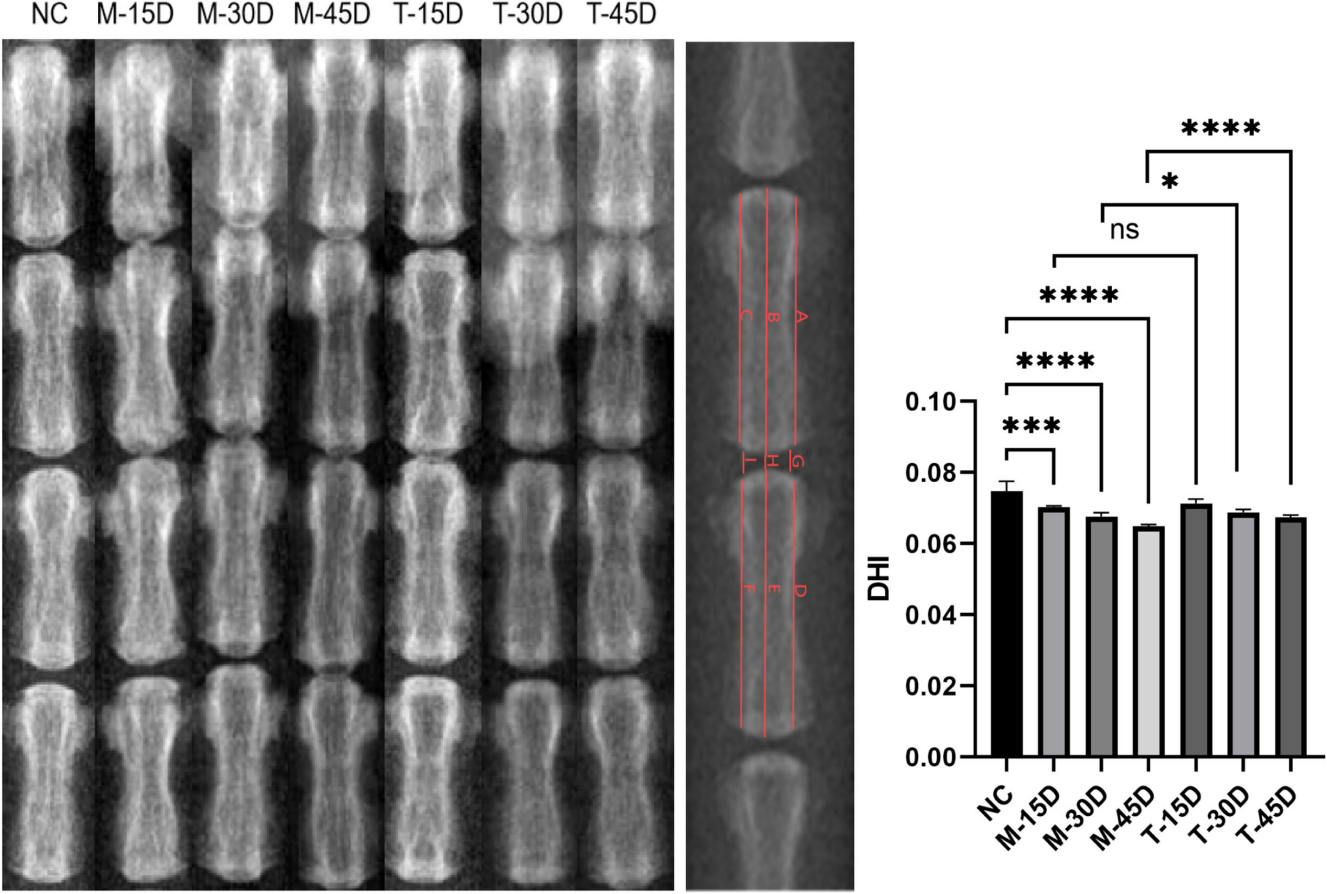
DR images and quantitative analysis of DHI in rat caudal intervertebral discs. Representative lateral DR images of the tail vertebrae from the blank control group (NC), model groups (M-15D, M-30D, M-45D), and treatment groups (T-15D, T-30D, T-45D). Data are presented as mean ± SD. Statistical comparisons were performed using one-way ANOVA followed by Tukey’s *post hoc* test. *p < 0.05; ***p < 0.001; ****p < 0.0001; ns, not significant.

Digital radiography (DR) analysis: X-rays were performed separately on all groups of rats at the end of gavage. Briefly, after anesthesia (same method as above), the rats were fixed on top of cardboard so that the tail was in a straight line. Images were then acquired using a DR instrument with orthostatic parameters at 80 kV for 120 ms and lateral parameters at 100 kV for 160 ms. The images were then analyzed and processed using ImageJ Pro. The disc height index (DHI) was used to determine the degree of disc degeneration. DHI = 2*(G+H+I)/(A+B+C+D+E+F); the illustration is shown in **Figure 1**.

Hematoxylin and eosin staining (HE): The rats in each group were anesthetized (same method as above) and then executed after removing their tails. The tailbones of rats were submerged in 4% paraformaldehyde for 48 hours and then immersed in ethylenediaminetetraacetic acid decalcification solution. Sealed and placed in a thermostatic shaker. The decalcification solution was changed daily for 1 month. Then embedded and sectioned. After microscopic examination, images were captured.

Masson’s trichrome staining (Masson): Rat tails were harvested, decalcified, embedded, and sectioned (steps as above). After microscopic examination, images were captured.

Fluorescence staining: Paraffin sections were subjected to antigenic repair. Sections were added to the trypsin repair solution and placed in an oven at 37 °C for 20 minutes. Sections were placed in an autoclave containing citrate buffer (pH 6) repair solution, heated to boiling, and left to cool. Sections were slightly dried and closed with 5% goat serum at ordinary temperature for 30 minutes. After closure, primary antibodies were added dropwise to the sections and incubated overnight. The slices were incubated for 60 min at ordinary temperature, protected from light, then dried and incubated for 5 minutes at ordinary temperature, protected from light, with a drop of DAPI staining solution and incubated for 5 minutes at ordinary temperature, protected from light, with a drop of tissue autofluorescence quencher b. After each of the above steps, the slices were washed three times, each time for 5 minutes. The slices were then sealed, examined microscopically, and imaged.

Histological scoring: The severity of IVDD was assessed in sections according to the histopathological grading system established by the orthopedic research society (ORS) spine section (29). Briefly, three independent observers blinded to the group assignments evaluated each disc. The final score for each disc was calculated as the mean of the three observers’ scores.

Immunofluorescence quantification: The mean fluorescence intensity (MFI) of aggrecan and collagen 2 was quantified using ImageJ Pro software (NIH, USA). Briefly, five randomly selected fields of view per section were analyzed, and the MFI was calculated after background subtraction. The percentage of M1 macrophages (CD68 and CD86) and M2 macrophages (CD68 and CD163) was calculated as the number of double-positive cells divided by the total number of DAPI-stained nuclei, expressed as a percentage. All quantifications were performed by two independent observers blinded to the group assignments.

#### 2.3.5 Western blot

Protein sample preparation. Rat tissue samples were taken, labeled, grouped, and added to each tube at a ratio of 1:10 rat disc and NP tissue mass (mg): RIPA lysate. The tissues were first minced using a sterile tissue slicer and placed in sterile, enzyme-free EP tubes, then lysed with an ultrasonic device and placed on crushed ice for 30 minutes. The EP tubes were then placed in a 4 °C centrifuge and centrifuged at 12,000 rpm for 15 minutes. The supernatant was taken, and the protein concentration was determined using the BCA Protein Quantification Kit.

Western blotting experiment. Each group was loaded with the corresponding samples according to the protein concentration, followed by electrophoresis with a 12% SD-PAGE gel and transfer of proteins with PVDF membranes. It was closed with 5% skimmed milk powder for 1 hour, and the membrane was washed by TBST. The primary antibody was incubated at 4 °C overnight, the membrane was washed by TBST, the second antibody was incubated at room temperature (37 °C) for 1 hour, the membrane was washed by TBST, and the corresponding chemiluminescence was added. Images were exported, and the gray values of the bands were analyzed by ImageJ Pro.

### 2.4 *In vitro* experiments

#### 2.4.1 Preparation of DHJSD-containing sera

Forty 3-month-old SD rats were randomly divided into two groups: the blank control group and the DHJSD gavage group. Converted the equivalent dose of DHJSD according to human and animal body mass ratio. The DHJSD group was treated with 1.7 g/kg by gavage, while the blank control group was given an equivalent amount of saline by gavage. Gavage was performed for 7 consecutive days, with 2 gavages on the last day at 2-hour intervals. Fast for 8 hours before blood collection. The blood was collected from the abdominal aorta under anesthesia 1 hour after the last gavage. The blood was kept in a 37 °C water bath for 30 minutes. After being centrifuged at 3000 rpm for 15 minutes, the upper serum layer was aspirated and then filtered through a 0.22 μm membrane for 30 minutes in a water bath at 56 °C for sterilization and then divided into portions and preserved in liquid nitrogen at -196 °C for spare use.

#### 2.4.2 Macrophage polarization and co-culture

Macrophage polarization, induction, and co-culture: THP-1 cells were inoculated in 6-well plates with serum-free medium and treated with 100 ng/mL phorbol 12-myristate 13-acetate (PMA) for 24 hours to achieve M0 polarization. Thereafter, 100 ng/ml lipopolysaccharide (LPS) + 50 ng/ml interferon-gamma (IFN-γ) in serum-free medium was treated for 24 hours to achieve M1 polarization, and 50 ng/ml interleukin-4 (IL-4) + 50 ng/ml interleukin-13 (IL-13) was treated for 24 hours to achieve M2 polarization. After polarization, trypsin digestion of half of the cells was used to identify markers by flow cytometry to determine the success of polarization. Take the other half of the cells, replace the medium with serum-free medium, and incubate for another 24 hours before harvesting the supernatant. The harvested supernatants were centrifuged and defined as macrophage-derived conditioned media, M1CM and M2CM, respectively. In addition, the same procedure as described above was performed by adding 50% DHJSD-containing serum to the culture medium of M0 macrophages at the time of the polarization process. Flow cytometry was then performed to identify the cell markers, while the conditioned media were collected as M1CM+DHJSD and M2CM+DHJSD, respectively. We defined the above subgroups as the NC group, M group, M1CM group, M2CM group, M1CM+DHJSD group, and M2CM+DHJSD group, respectively.

M1, M2 macrophage identification: Polarized M1 macrophage markers (CD68+CD86) and M2 macrophage markers (CD68+CD163) were detected separately using flow cytometry.

Co-culture of NPCs with macrophages: The NPCs were divided into 6 groups: 10% FBS medium is the blank control group (NC group), 100 ug/ml LPS is the negative group (M group), 100 ug/ml LPS and 50% M1CM is the co-culture group (M+M1CM group), 100 ug/ml LPS and 50% M2CM is the co-culture group (M+M2CM group), 100 ug/ml LPS and 50% M1CM+DHJSD is the co-culture group (M+M1CM+DHJSD group), and 100 ug/ml LPS and 50% M2CM+DHJSD is the co-culture group (M+M2CM+DHJSD group).

Western blotting: After the cells were co-cultured for 24 hours, cell-extracted proteins were collected from each group, and Western blotting experiments were performed (same method as above).

Statistical analysis: Statistical analyses were performed using Graphpad Prism 7, and the data were expressed as mean ± standard deviation (x¯ ± s). For comparisons involving only two groups, an unpaired Student’s t-test was used. For comparisons involving three or more groups, one-way analysis of variance (ANOVA) followed by Tukey’s *post hoc* test for multiple comparisons was applied. Differences were considered statistically significant at P < 0.05. All quantitative results were calculated from at least three biological replicates.

## 3 Results

### 3.1 Single-cell sequencing data

**Figure 2:**
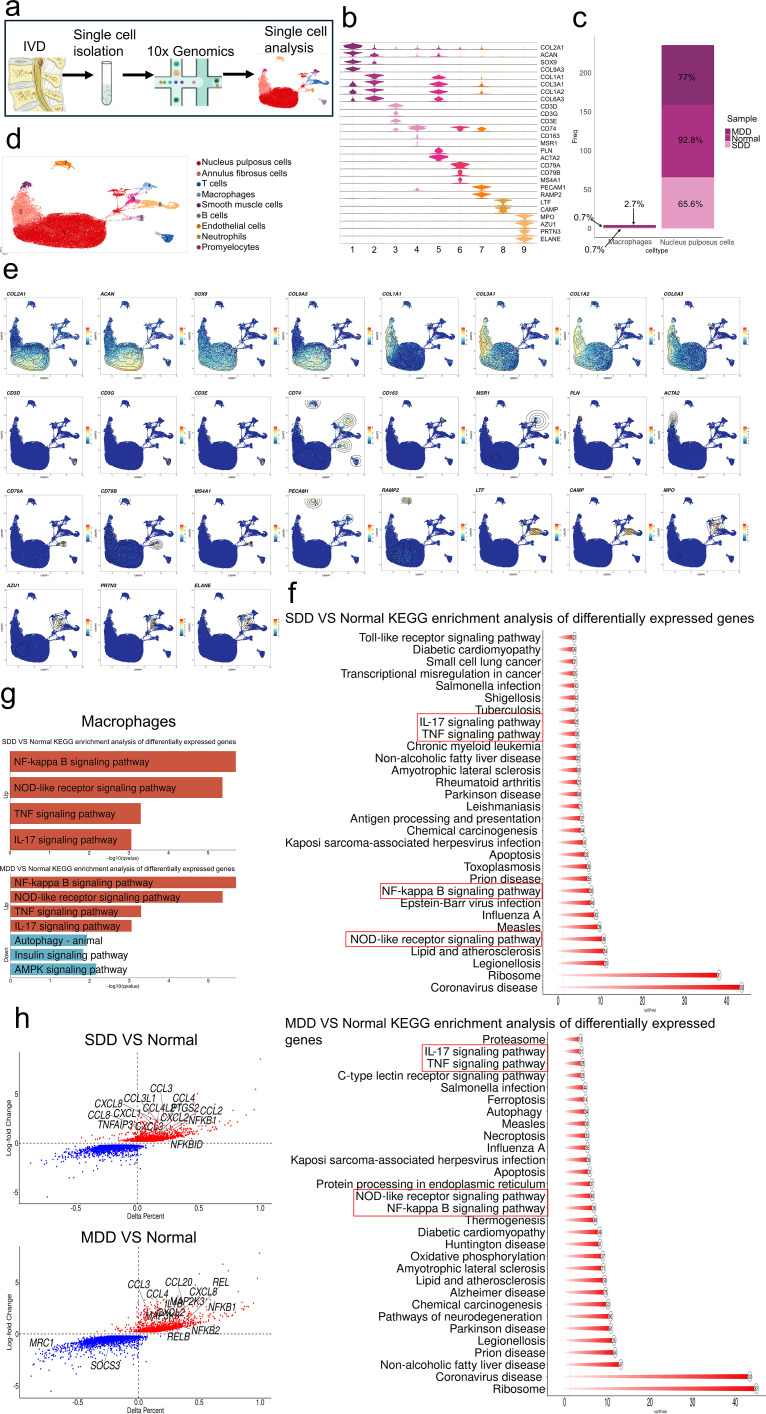
Macrophages are the primary cells driving the immune-inflammatory response in IVDD. **(a)** Schematic overview of single-cell sequencing workflow. **(b)** Violin plots showing expression levels of marker genes across nine annotated cell types. **(c)** Bar graph showing the percentage of macrophages and NPCs in each group. **(d)** UMAP plot of all 57,476 cells from IVDD and normal samples, colored by cell type (NPCs, fibroblasts, T cells, macrophages, smooth muscle cells, B cells, endothelial cells, neutrophils, and promyelocytes). **(e)** UMAP contour plots showing expression of representative marker genes for each cell type (blue: low expression; yellow/red: high expression). **(f)** KEGG enrichment analysis of differentially expressed genes between the SDD group and the blank control group, and between the MDD group and the blank control group. **(g)** KEGG enrichment analysis of differentially expressed genes in macrophages, with upregulated pro-inflammatory pathways shown in red and downregulated anti-inflammatory pathways shown in blue. **(h)** Heatmap of differentially expressed genes in macrophages between SDD/MDD groups and the blank control group (red: upregulated; blue: downregulated).

To further investigate the specific role of macrophages in the development of IVDD, we used a combined analysis of seven IVDD patient samples from GSE244889 and one normal disc tissue sample from GSE205535 (**Figure 2a**). We obtained 57,476 cells and used the Find-All-Markers function in R to identify genes specific to different cell populations. We annotated nine different cell types: NPCs, fibroblasts, T cells, macrophages, smooth muscle cells, B cells, endothelial cells, neutrophils, and promyelocytes. We found that a decrease in the proportion of NPCs in IVDD is accompanied by an increase in the proportion of macrophages (**Figures 2b–e**). We then performed differential analysis of the severe disc degeneration (SDD) group versus the black control group and the moderate disc degeneration (MDD) group versus the black control group and performed KEGG enrichment analysis with the obtained differential genes, both of which revealed that the immune-inflammation-related pathways were significantly up-regulated in SDD and MDD (**Figure 2f**). We performed enrichment analysis of differential genes in different groups of macrophages, and immune-inflammation-related pathways were similarly enriched, and down-regulation of immune-inflammation-related pathways that are negatively regulated was also enriched in MDD (**Figure 2g**). Many of the genes associated with the promotion of inflammation were also up-regulated among the differential genes in different groups of macrophages, and down-regulation was seen in genes associated with M2 macrophage polarization and anti-inflammatory genes (**Figure 2h**). These results suggest that macrophages are the major cells triggering the immune-inflammatory response in IVDD.

**Figure 3:**
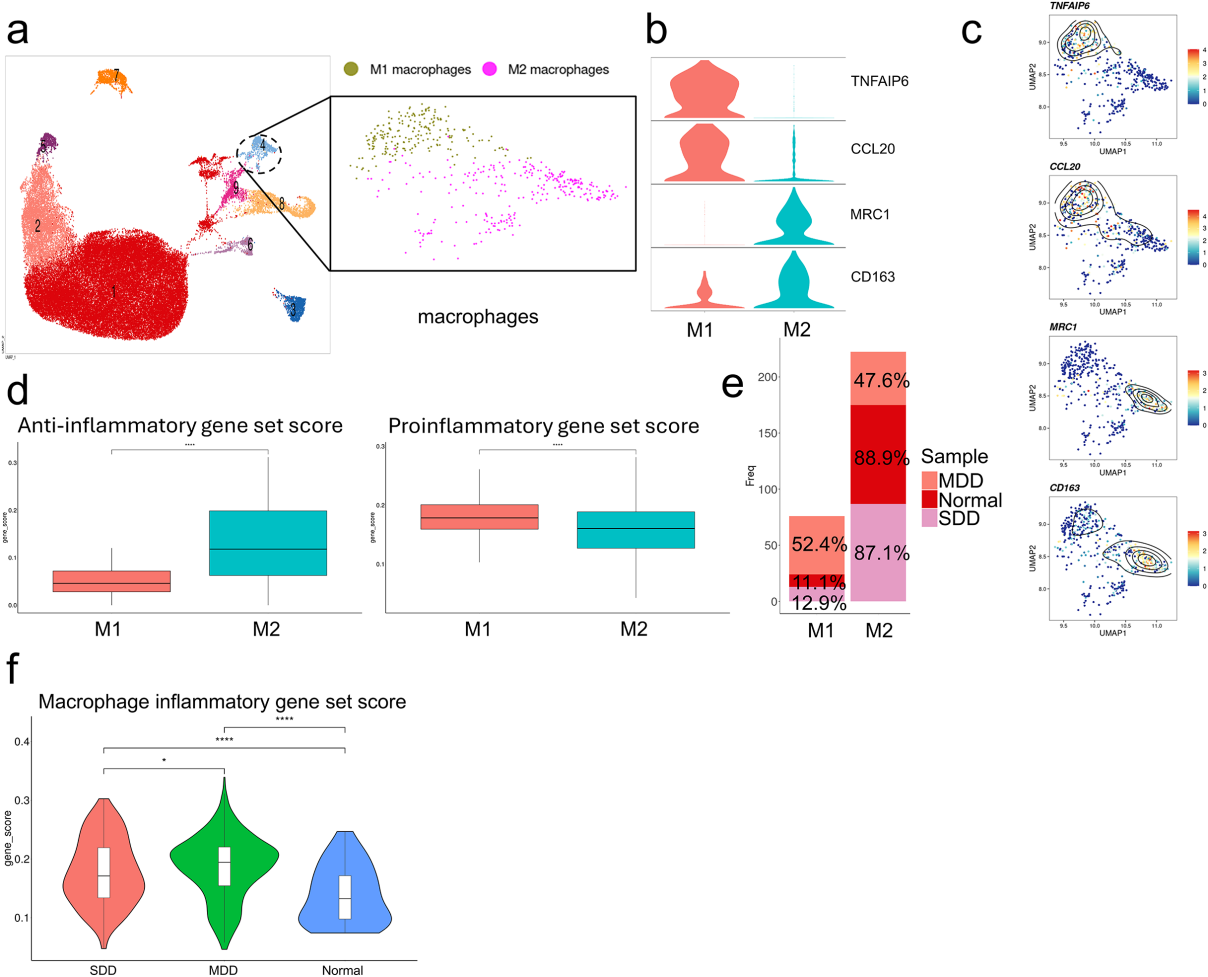
Increased M1 macrophage polarization in patients with IVDD. **(a)** UMAP plot showing M1 and M2 macrophage clusters identified from all macrophage samples. **(b)** Violin plots showing expression levels of representative marker genes for M1 (CD86) and M2 (CD163) macrophages. **(c)** UMAP contour plots showing expression of CD86 and CD163 across the two macrophage subtypes. **(d)** Box plots showing anti-inflammatory and pro-inflammatory gene set scores in M1 versus M2 macrophages. **(e)** Bar graph showing the proportion of M1 and M2 macrophages in each sample group (blank control, MDD, SDD). **(f)** Pro-inflammatory gene set scores of macrophages from the blank control, MDD, and SDD groups. n = 7 IVDD samples and 1 normal sample for single-cell analysis. *p < 0.05, ****p < 0.0001.

To further look for changes in different subpopulations of macrophages in patients, we selected macrophages, clustered them into subgroups, and labeled them as M1 and M2 macrophages based on representative genes (**Figures 3a–c**). Macrophages after clustering were scored using anti-inflammatory and inflammatory gene sets, respectively. The results showed that the secretion of anti-inflammatory factors was significantly higher in cells labeled as M2 macrophages compared to M1 macrophages, while the secretion of inflammatory factors was significantly higher in cells labeled as M1 macrophages compared to M2 macrophages (**Figure 3d**). Notably, the proportion of M1-type macrophages was elevated in macrophages from patients with IVDD, and the inflammatory gene set score also suggested elevated levels of inflammation in macrophages in the SDD and MDD groups (**Figures 3e, f**). Therefore, we hypothesized that in IVDD, more macrophages polarized to the M1 type exacerbated the immune inflammatory response and that inhibiting M1 macrophage polarization and promoting M2 cell polarization could effectively ameliorate the immune inflammatory response and ameliorate IVDD.

### 3.2 Bioinformatics data

**Figure 4:**
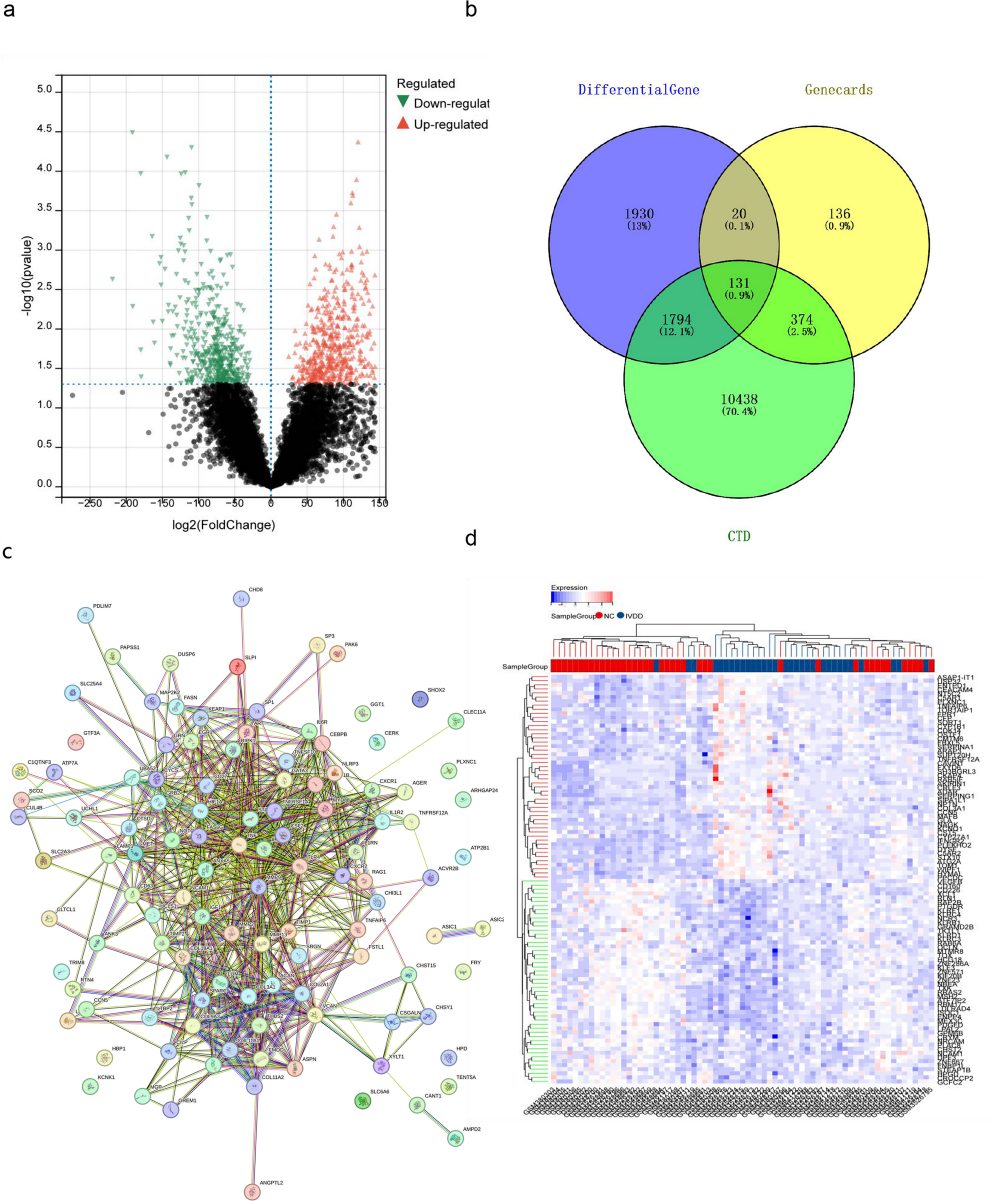
Identification of key genes associated with IVDD. **(a)** Volcano plot showing DEGs between IVDD samples (n = 31) and normal samples (n = 40) from the combined GSE15227, GSE34095, GSE124272, and GSE150408 datasets. Red dots represent significantly upregulated genes; blue dots represent significantly downregulated genes. **(b)** Venn diagram showing the intersection of DEGs from the four GEO datasets with IVDD-associated genes from the GeneCards and CTD, yielding 131 common genes. **(c)** PPI network of the 131 intersecting genes, generated using STRING database with a confidence score > 0.4. **(d)** Heatmap showing expression patterns of the top 50 intersecting genes in IVDD samples versus normal samples. Red, high expression; blue, low expression.

After data processing of GSE15227, GSE34095, GSE124272, and GSE150408 downloaded from the GEO database, the remaining 71 samples included 31 IVDD samples and 40 normal samples. Data were transformed by gene ID to obtain a dataset containing gene names. The results of the differential volcano map showed that out of 12,322 genes, 1,227 were differential, of which 753 genes were upregulated and 474 genes were downregulated (**Figure 4a**). Downloaded through different databases, 661 genes were from GeneCard, and 12,737 genes were from CTD. The sum of the GSE15227, GSE34095, GSE124272, and GSE150408 differential genes was overlapped with the GeneCard and CTD data, and we got 131 intersections (**Figure 4b**). The PPI network shows that these 131 crossover genes interact with each other, and the differential heatmap shows the differential expression of the top 50 genes of the crossover genes (**Figures 4c,d**).

**Figure 5:**
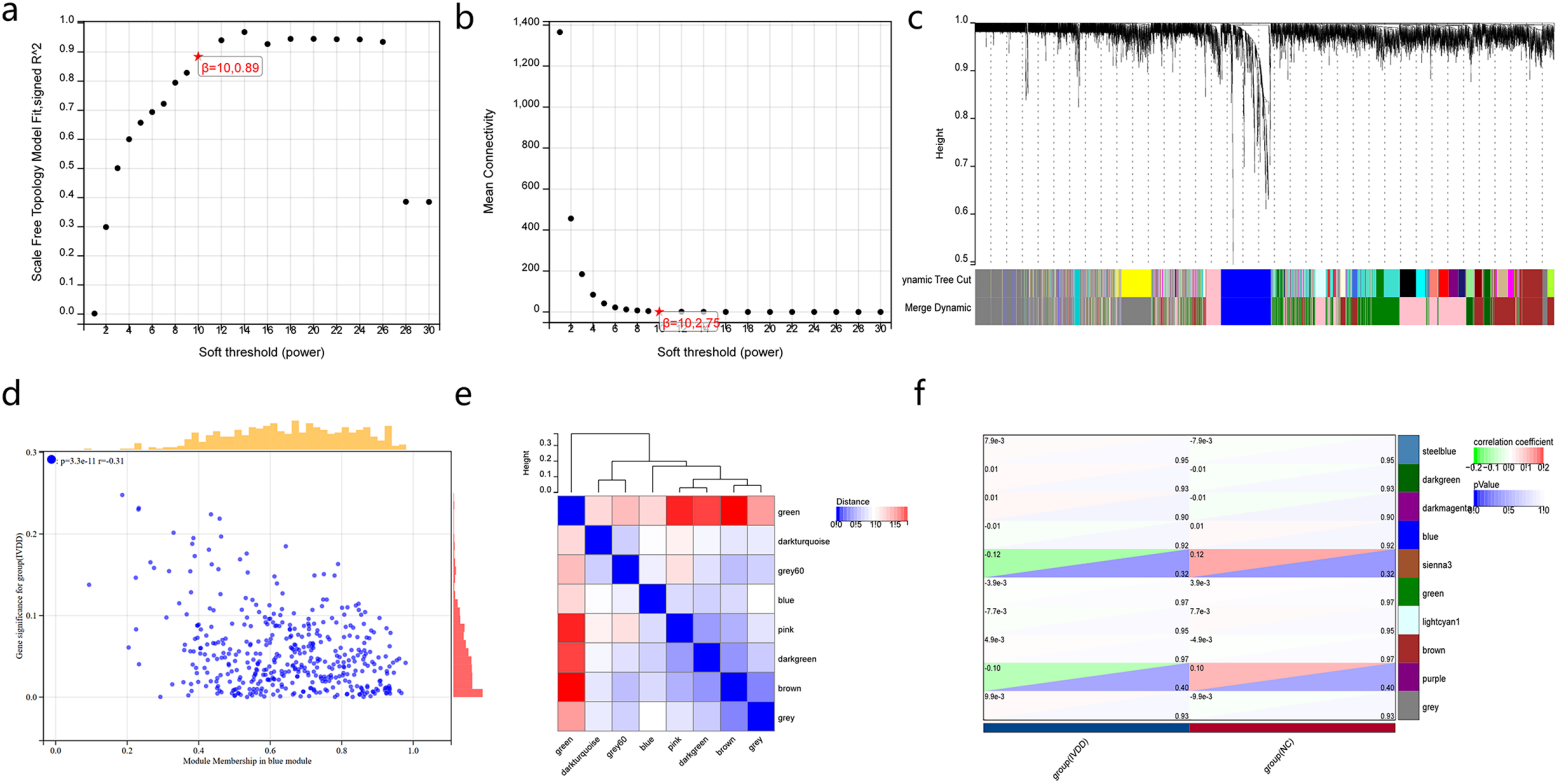
WGCNA of IVDD-associated modules. **(a)** Scale-free topology model fit (R²) at different soft-thresholding powers. A soft threshold of 10 (R² = 0.89) was selected for network construction. **(b)** Mean connectivity at different soft-thresholding powers. **(c)** Hierarchical clustering dendrogram of genes based on topological overlap, with modules identified by dynamic tree cutting (minimum module size=30). **(d)** Correlation analysis between MM and GS for the green module. **(e)** Clustering dendrogram of module eigengenes, showing the relationship among identified modules. **(f)** Module-trait correlation heatmap showing the correlation between each module and IVDD phenotype.

After removing the abnormal samples and screening genes, the expression profiles of 12,232 copies of the GSE15227, GSE34095, GSE124272, and GSE150408 samples were extracted to construct weighted gene co-expression networks. When the soft threshold power was set to 10, the scale independence reached 0.89 and the average connection value was 102.75 (**Figures 5a, b**). When the cutting height was set to 0.25 and the minimum module size was set to 30, 8 different co-expression modules were obtained by dynamic tree cutting (**Figure 5e**). Vector clustering analysis for each module showed that green has the greatest distance from the blue module (**Figure 5c**). The modules were correlated with clinical characteristics. The green module in ME was positively correlated with IVDD (correlation coefficient = 3.9e^-3^; p = 0.97), and the ME blue module was positively correlated with IVDD (correlation coefficient = 0.01; p = 0.92; **Figure 5f**). In addition, correlation analyses of MM and GS showed that these genes were highly correlated with modules and phenotypes (r = -0.31, p = 3.3e^-11^, **Figure 5d**).

**Figure 6:**
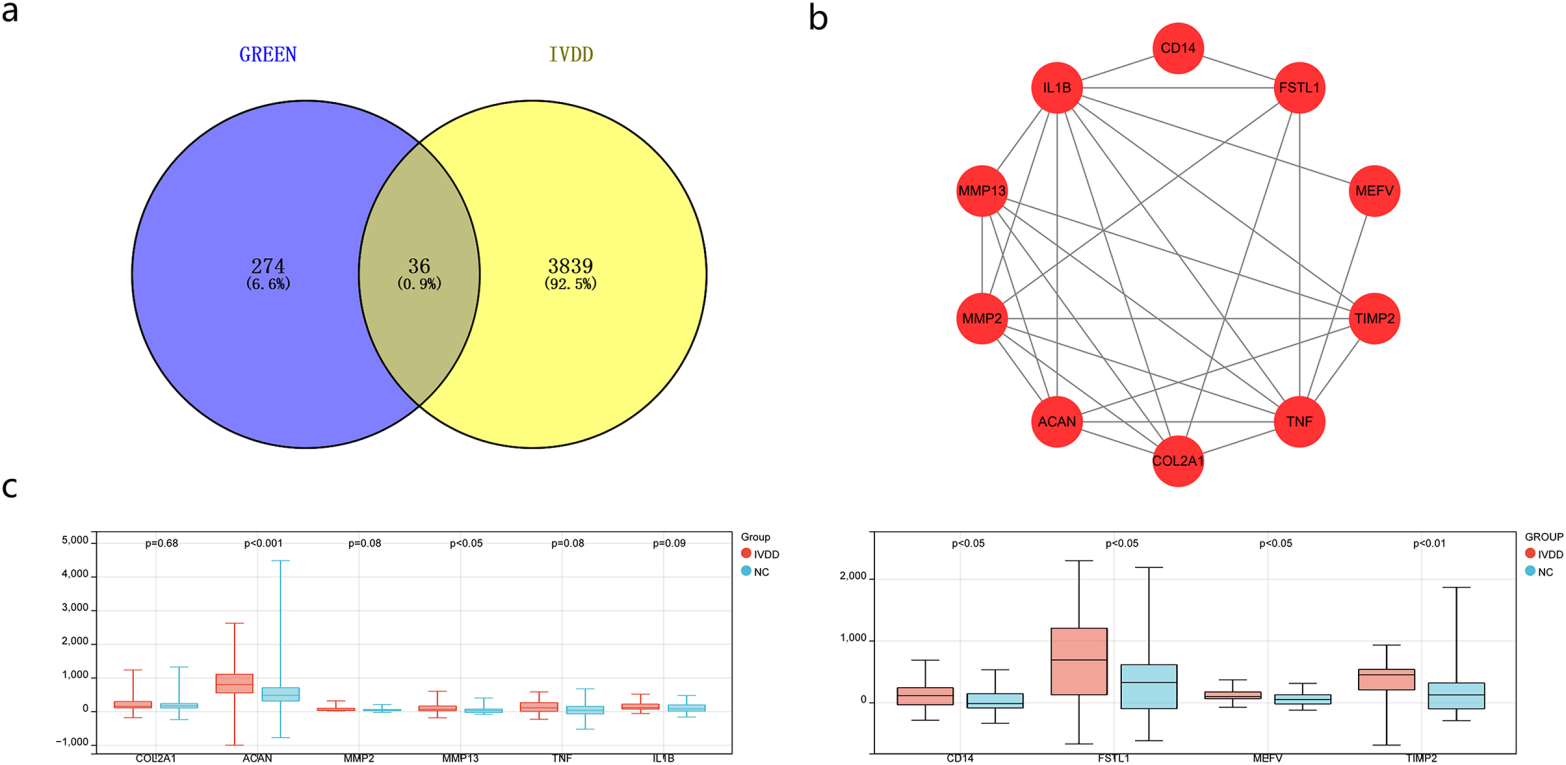
Identification and validation of hub genes for IVDD. **(a)** Venn diagram showing the overlap between the green module genes from WGCNA (310 genes) and the 131 IVDD-associated genes identified in **Figure 4b**, yielding 36 candidate key genes. **(b)** Protein-protein interaction network of the top 10 hub genes identified using CytoHubba (MCC algorithm) in Cytoscape. **(c)** Box plots showing expression levels of the 10 hub genes in IVDD samples versus normal samples.

WGCNA showed that green was highly correlated with the clinical features of IVDD and that green contained 310 genes. Green again intersected with 3875 crossover genes and obtained 36 key genes (**Figure 6a**). After calculation, we obtained the 10 hub genes (**Figure 6b**), and the differential expression analysis of collagen type II alpha 1 chain (COL2A1), aggrecan (ACAN), MMP2, MMP13, TNF, IL-1β, cluster of differentiation 14 (CD14), follistatin-like 1 (FSTL1), Mediterranean fever (MEFV), and tissue inhibitor of metalloproteinases 2 (TIMP2) showed statistical significance for ACAN, MMP13, CD14, FSLT1, MEFV, and TIMP2 (**Figure 6c**).

### 3.3 *In vivo* experimental data

#### 3.3.1 DHJSD mass spectrometry

**Figure 7:**
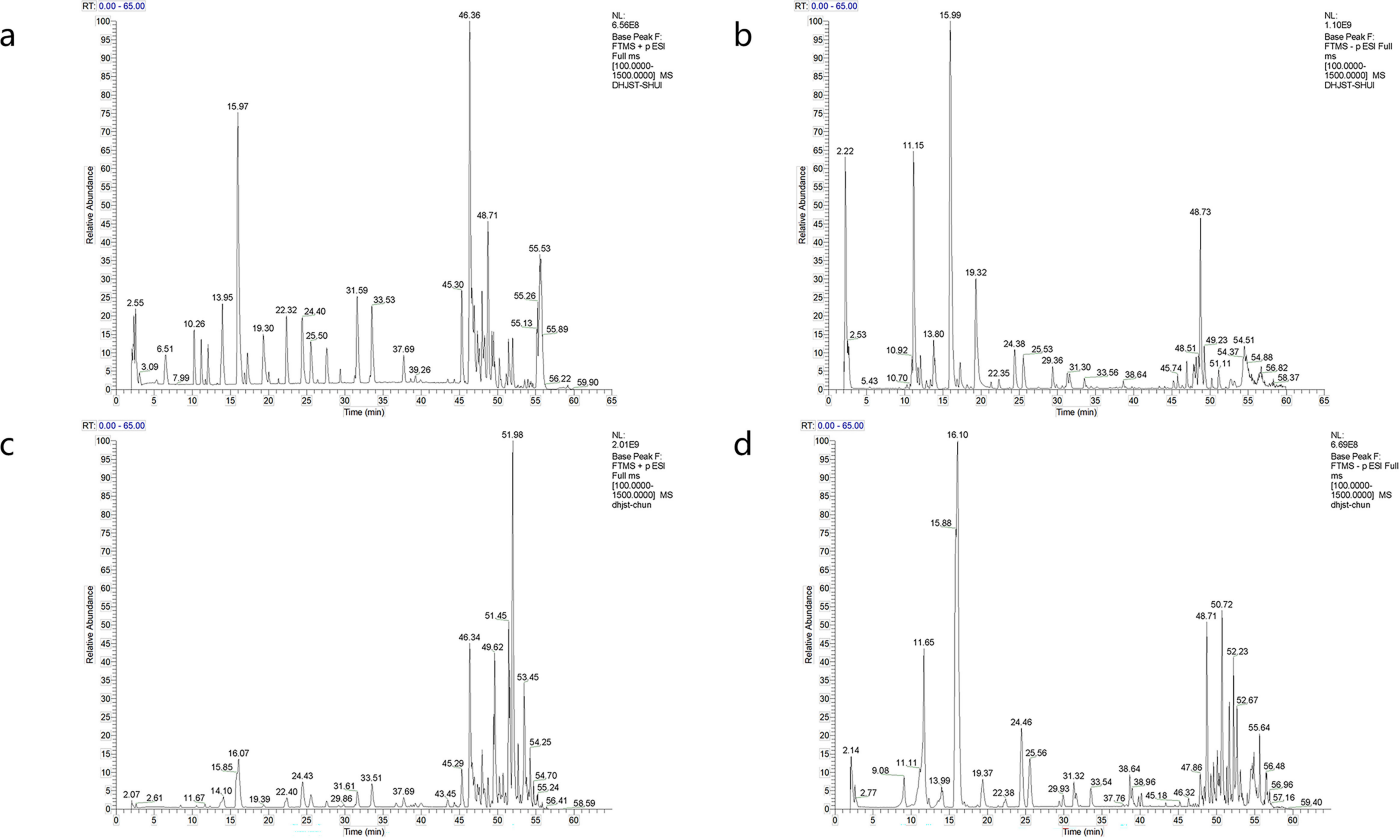
Mass spectrometry analysis of DHJSD. **(a)** Total ion chromatogram of water-extracted DHJSD in positive ion mode. **(b)** Total ion chromatogram of water-extracted DHJSD in negative ion mode. **(c)** Total ion chromatogram of ethanol-extracted DHJSD in positive ion mode. **(d)** Total ion chromatogram of ethanol-extracted DHJSD in negative ion mode. The active pharmaceutical ingredients identified from each extract are listed in **Supplementary Table 2** (water extract) and 3 (ethanol extract). Analysis was performed using a Q Exactive Mass Spectrometer, and peaks were annotated by comparison with reference standards and the herbal ingredient database. Representative chromatograms from three independent runs are shown.

As shown in **Figure 7**, we performed mass spectrometry analysis of DHJSD to obtain the active ingredients therein. The active pharmaceutical ingredients purified in water from DHJSD are shown in **Supplementary Table 2**. The active pharmaceutical ingredients purified in ethanol from DHJSD are shown in **Supplementary Table 3**.

#### 3.3.2 Imaging and histomorphological findings

**Figure 8:**
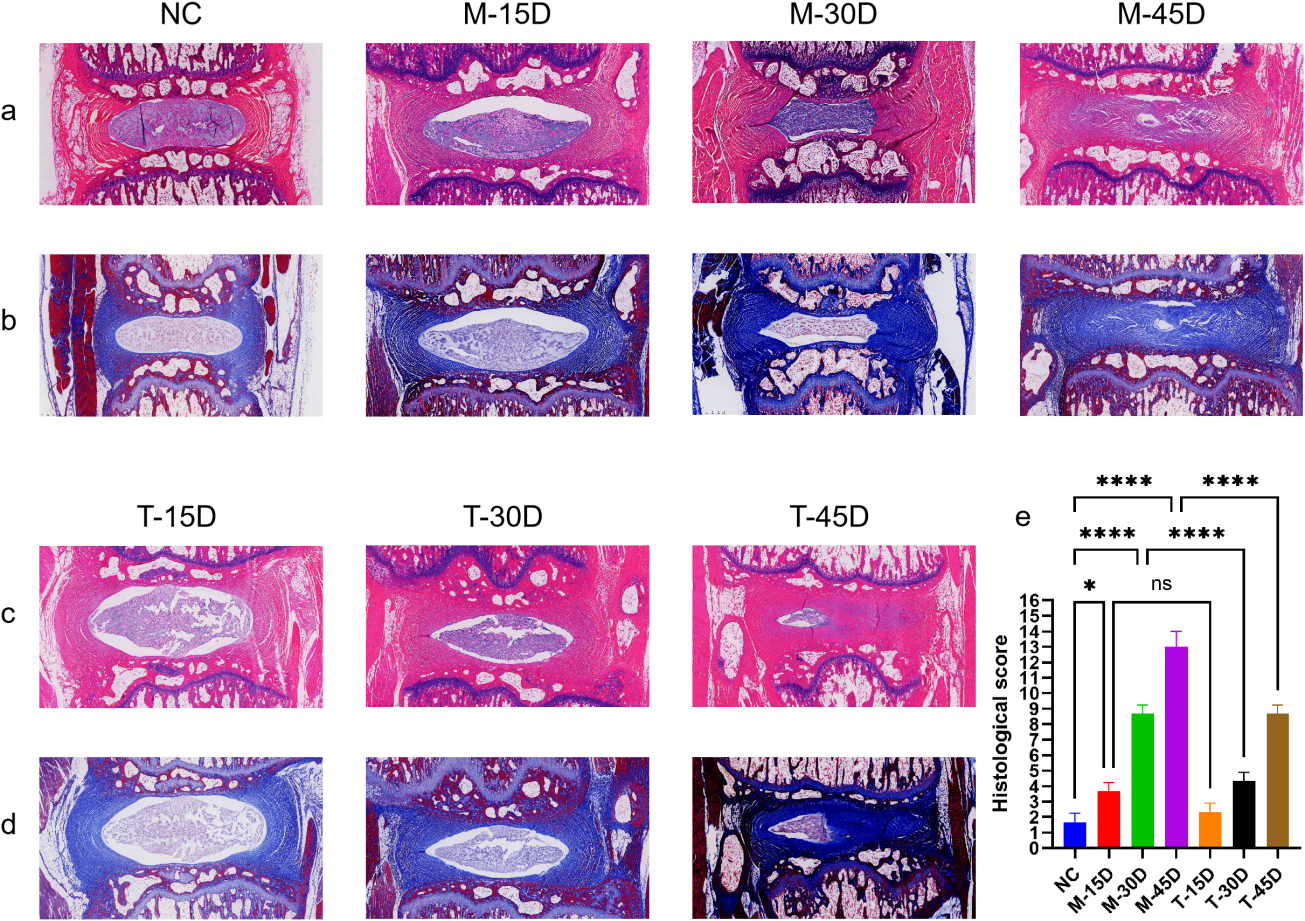
Histological assessment of IVDD. **(a)** Representative HE-stained sections of the blank control group (NC) and model groups (M-15D, M-30D, M-45D). **(b)** Representative Masson-stained sections of the NC and model groups. **(c)** Representative HE-stained sections of the treatment groups (T-15D, T-30D, T-45D) compared with the corresponding model groups. **(d)** Representative Masson-stained sections of the treatment groups compared with the corresponding model groups. **(e)** Quantitative analysis of histological scores based on the ORS spine grading system. Data are presented as mean ± SD. Statistical comparisons were performed using one-way ANOVA followed by Tukey’s *post hoc* test. *p < 0.05, ****p < 0.0001; ns, not significant.

**Figure 9:**
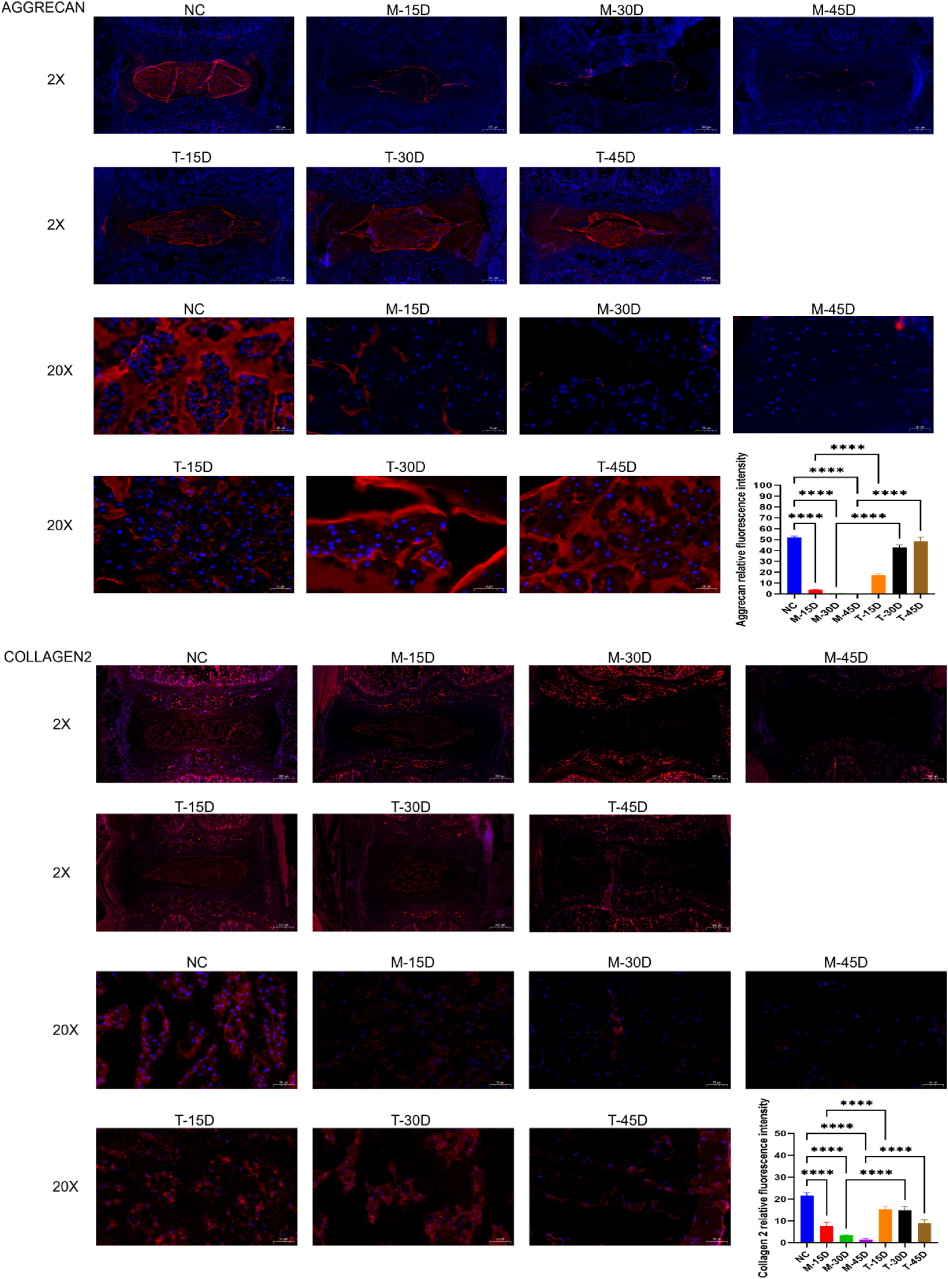
Immunofluorescence staining and quantitative analysis of ECM proteins. Representative immunofluorescence images showing aggrecan and collagen 2 expression in the nucleus pulposus from the blank control group (NC), model groups (M-15D, M-30D, M-45D), and treatment groups (T-15D, T-30D, T-45D). The MFI of aggrecan and collagen 2 was quantified using ImageJ Pro. Data are presented as mean ± SD. Statistical comparisons were performed using one-way ANOVA followed by Tukey’s *post hoc* test. ****p < 0.0001.

DR images demonstrate that the model group’s vertebral body height was considerably lower than that of the NC group and that the disc height stenosis grew noticeably as modeling time rose. When comparing the T-30D and T-45D groups to the same timepoint model group, there was a noticeable increase in vertebral height (**Figure 1**). Histological evaluation was performed using HE and Masson staining. The degree of IVDD was assessed using the histopathological grading system established by the ORS spine section (29). As shown in **Figures 8a, b**, the histological scores in the three model groups were significantly higher than those in the NC group (p < 0.05) and the histological scores of the three model groups gradually increased, indicating progressive degeneration over time. Compared with the corresponding model groups, DHJSD treatment significantly reduced the histological scores in the T-30D and T-45D groups, whereas no significant difference was observed between the T-15D and M-15D groups (p > 0.05) (**Figures 8c, d**). Immunofluorescence staining of tissue sections showed that compared with the NC group, the MFI of both aggrecan and collagen 2 was significantly decreased in the three model groups (p < 0.05), and the degree of reduction of the ECM increased with the prolongation of time after modeling. DHJSD treatment significantly increased the MFI of both ECM proteins in all treatment groups compared with the corresponding model groups (p < 0.05) (**Figure 9**). This indicates that the IVDD model was successfully established by needling the caudal intervertebral disc of rats, and DHJSD can effectively ameliorate IVDD. The therapeutic effect of DHJSD gavage at 30 and 45 days after modeling was significantly better than that of gavage at 15 days after modeling. We can conclude that DHJSD can ameliorate IVDD, and the therapeutic effect of DHJSD is better in the middle and late stages of IVDD.

#### 3.3.3 Expression of M1 and M2 type macrophages in black control and model rat tissues

**Figure 10:**
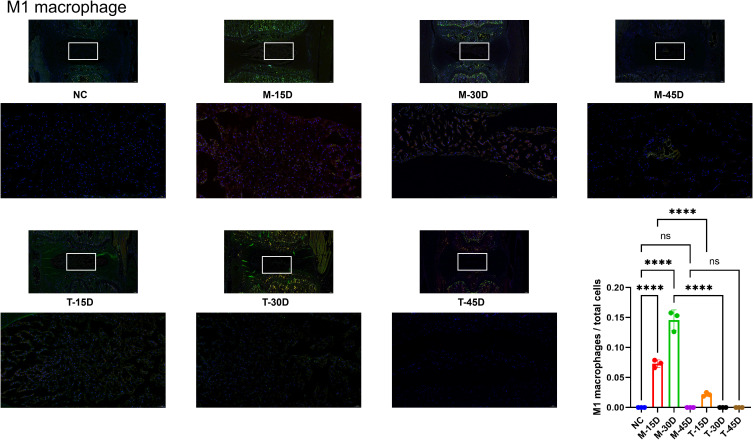
Immunofluorescence staining and quantitative analysis of M1 macrophages. Representative immunofluorescence images showing M1 macrophages co-expressing CD68 (green) and CD86 (red) in the nucleus pulposus from the blank control group (NC), model groups (M-15D, M-30D, M-45D), and treatment groups (T-15D, T-30D, T-45D). Nuclei were counterstained with DAPI (blue). M1 macrophages were quantified as the percentage of CD68^+^ and CD86^+^ cells relative to total DAPI-stained nuclei. Data are presented as mean ± SD. Statistical comparisons were performed using one-way ANOVA followed by Tukey’s *post hoc* test. ****p < 0.0001; ns, not significant.

**Figure 11:**
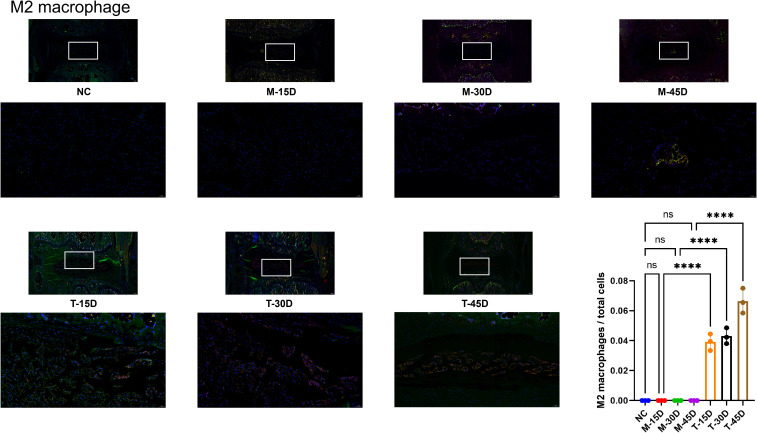
Immunofluorescence staining and quantitative analysis of M2 macrophages. Representative immunofluorescence images showing M2 macrophages co-expressing CD68 (green) and CD163 (red) in the nucleus pulposus from the blank control group (NC), model groups (M-15D, M-30D, M-45D), and treatment groups (T-15D, T-30D, T-45D). Nuclei were counterstained with DAPI (blue). M2 macrophages were quantified as the percentage of CD68 and CD163 cells relative to total DAPI-stained nuclei. Data are presented as mean ± SD. Statistical comparisons were performed using one-way ANOVA followed by Tukey’s *post hoc* test. ****p < 0.0001; ns, not significant.

As shown in **Figures 10**, **11**, to investigate whether macrophages are involved in the progression of IVDD and whether there is a temporal correlation, we performed immunofluorescence staining of the sample tissues. We applied CD68 (green) and CD86 (red) to label M1 macrophages in the sample and CD68 (green) and CD163 (red) to label M2 macrophages in the sample. The percentage of M1 macrophages was significantly higher in the M-15D and M-30D groups compared with the NC group. At 45 days post-modeling (M-45D), the NP region exhibited extensive cell loss, and M1 macrophages were rarely detectable (**Figure 10**). DHJSD treatment significantly reduced the percentage of M1 macrophages in the T-15D and T-30D groups compared with the corresponding model groups. Notably, M2 macrophages (CD68 and CD163) were absent in all model groups, indicating that endogenous M2 polarization does not occur during the natural progression of IVDD in this model. However, following DHJSD treatment, M2 macrophages became detectable in all treatment groups, with a striking temporal pattern: the percentage of M2 macrophages was highest in the T-45D group, followed by T-30D, and lowest in T-15D (**Figure 11**). We can conclude that macrophages are involved in the process of IVDD and that DHJSD is able to effectively regulate macrophage expression in IVDD model rats, leading to an amelioration of IVDD.

#### 3.3.4 DHJSD effectively reduces the degradation of ECM and inflammatory factors in a rat model of IVDD

**Figure 12:**
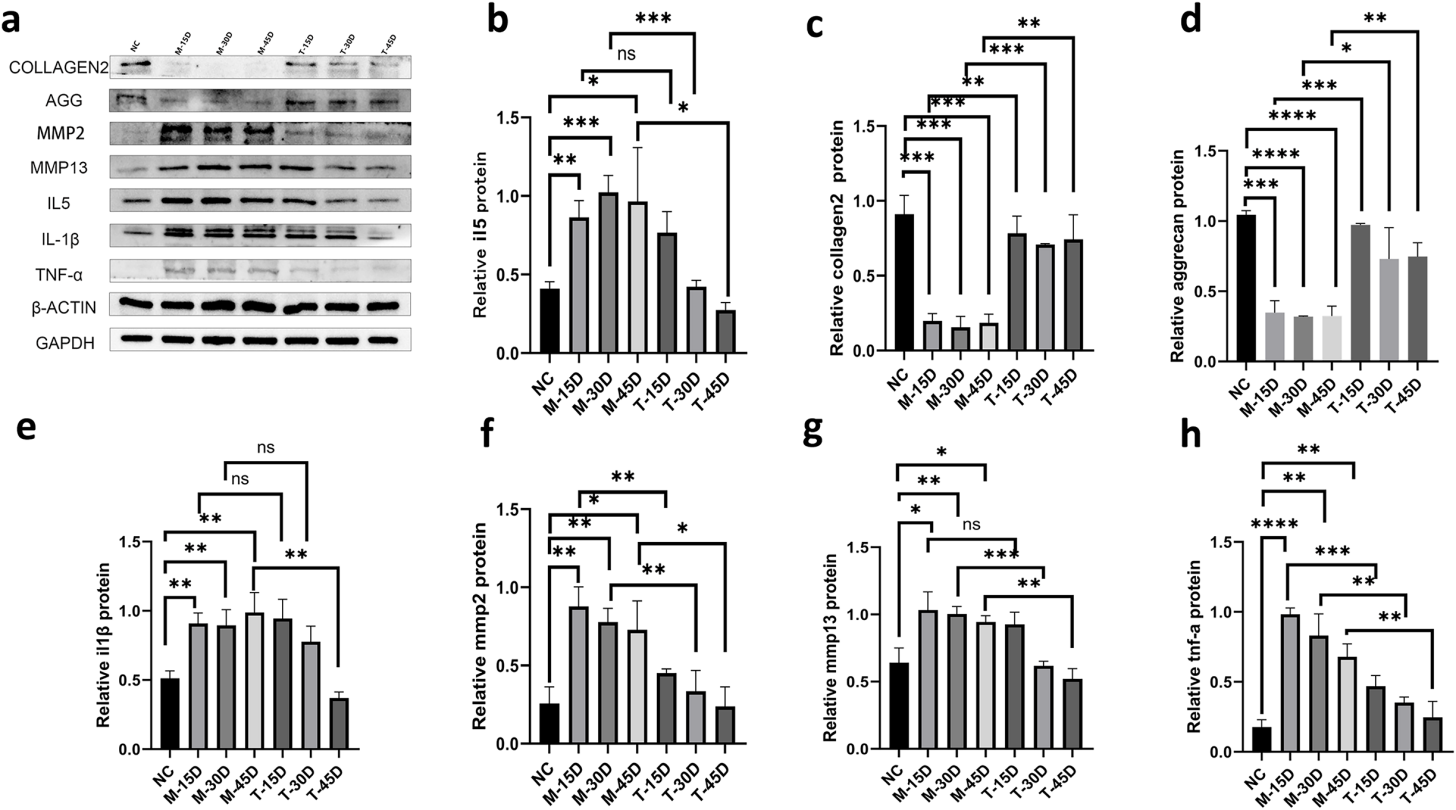
Effects of DHJSD on ECM degradation and inflammatory factor expression in a rat IVDD model. Representative Western blot bands of aggrecan, collagen 2, MMP13, MMP2, IL-5, IL-1β, and TNF-α protein expression in NP tissues from the blank control group (NC), model groups (M-15D, M-30D, M-45D), and treatment groups (T-15D, T-30D, T-45D) **(a)**. Quantitative analysis of aggrecan, collagen 2, MMP13, MMP2, IL-5, IL-1β, and TNF-α protein expression levels **(b–h)**. Data are presented as mean ± SD (n = 3 independent experiments). Statistical comparisons were performed using one-way ANOVA followed by Tukey’s *post hoc* test. *p < 0.05; **p < 0.01; ***p < 0.001; ****p < 0.0001; ns, not significant.

As shown in **Figure 12**, by Western blot analysis of the expression of ECM-associated proteins and related proteases, we found that aggrecan and collagen 2 were significantly lower in the model group compared to the black control group. The aggrecan and collagen 2 of the DHJSD treatment group were significantly better than those of the model group at the same time point, and the *in vivo* inflammatory factors IL-5, IL-1β, and TNF-α, as well as MMP13 and MMP2, showed a trend contrary to that of the ECM. These results suggest that DHJSD treatment can effectively reduce the secretion of inflammatory mediators and reduce the synthesis of relevant ECM catabolic proteins, thereby promoting ECM accumulation. According to the results, DHJSD gavage was performed 15 days after modeling, and the differences between the IL-5, IL-1β, and MMP13 treatment and model groups were not statistically significant. However, inflammatory factors, ECM proteins, and matrix metalloproteinases (MMPs) were statistically significant when T-30D and T-45D were compared with their counterparts, M-30D and M-45D, and the results showed a favorable effect of DHJSD. It can be concluded that DHJSD is effective in ameliorating IVDD and is better for the middle and late stages of IVDD.

### 3.4 *In vitro* experimental data

#### 3.4.1 Validation of M1 and M2 macrophage polarization

**Figure 13:**
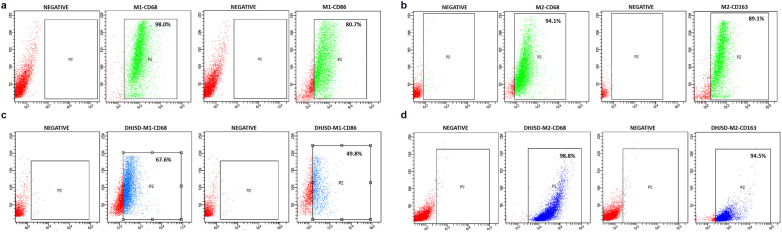
Flow cytometry validation of M1 and M2 macrophage polarization with and without DHJSD-containing serum treatment. **(a)** Representative flow cytometry plots showing M1 macrophage markers (CD68 and CD86) after induction with LPS + IFN-γ. **(b)** Representative flow cytometry plots showing M2 macrophage markers (CD68 and CD163) after induction with IL-4 + IL-13. **(c)** Representative flow cytometry plots showing M1 macrophage markers after treatment with 50% DHJSD-containing serum during the polarization process. **(d)** Representative flow cytometry plots showing M2 macrophage markers after treatment with 50% DHJSD-containing serum during the polarization process. Positive rates are indicated in the plots.

As shown in **Figure 13**, THP-1 cells were inoculated in 6-well plates and treated with PMA to achieve M0 polarization. M1 polarization was achieved using LPS and IFN-γ, and M2 polarization using IL-4 and IL-13. M1 macrophage markers and M2 macrophage markers indicating successful macrophage transformation were all detected using flow cytometry.

#### 3.4.2 Effect of macrophages on ECM and inflammatory mediator secretion in LPS-treated NPCs

**Figure 14:**
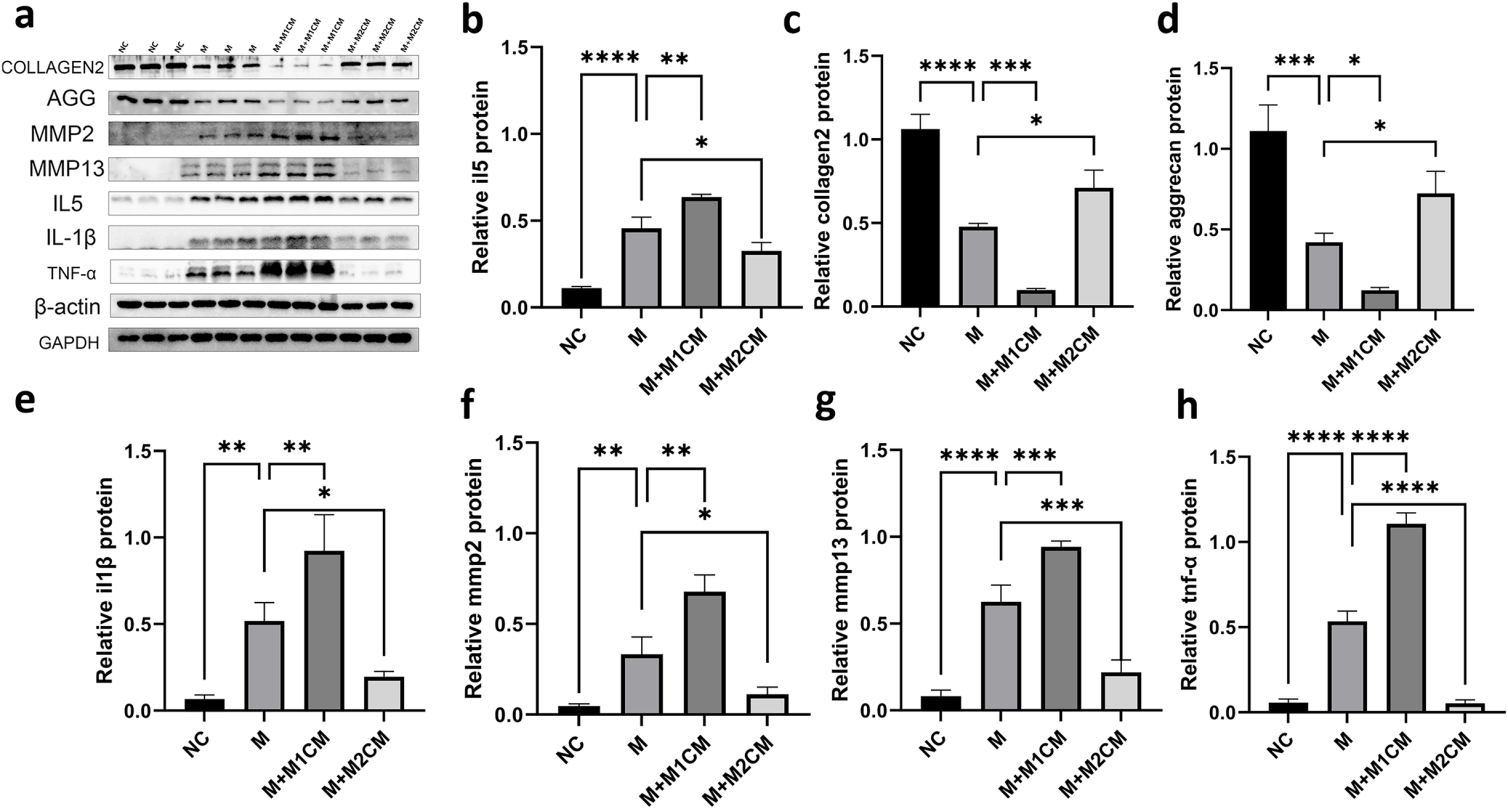
Effects of macrophage-conditioned media on ECM degradation and inflammatory factor expression in LPS-treated NPCs. Representative Western blot bands of aggrecan, collagen 2, MMP13, MMP2, IL-5, IL-1β, and TNF-α protein expression in NPCs under different co-culture conditions **(a)**. Quantitative analysis of aggrecan, collagen 2, MMP13, MMP2, IL-5, IL-1β, and TNF-α protein expression levels **(b–h)**. Data are presented as mean ± SD (n = 3 independent experiments). Statistical comparisons were performed using one-way ANOVA followed by Tukey’s *post hoc* test. *p < 0.05, **p < 0.01, ***p < 0.001, ****p < 0.0001.

First, we used Western blot to analyze the expression of ECM-associated proteins and associated proteases. We found that LPS treatment of NPCs significantly reduced the expression of ECM aggrecan and collagen 2. The M1CM co-culture group had significantly lower levels of aggrecan and collagen 2 than the LPS group, while the M2CM co-culture group had significantly higher levels of aggrecan and collagen 2 than the LPS group. In contrast, MMP13 and MMP2 showed an opposite trend, with MMP13 and MMP2 significantly higher in the M1CM co-culture group than in the LPS group and lower in the M2CM co-culture group than in the LPS group. By analyzing the expression of relevant inflammatory factors, we found that inflammatory factors such as TNF-α, IL-1β, and IL-5 were significantly higher in group M than in the black control group. Compared with the M group, TNF-α, IL-1β, and IL-5 were significantly higher in the M1CM group and significantly lower in the M2CM group (**Figures 14a–h**). These outcomes suggest that M1CM decreased ECM synthesis and facilitated ECM degradation in IVDD, as well as the secretion of inflammatory mediators, whereas M2CM was able to effectively decrease the secretion of inflammatory mediators and enhance the accumulation of ECM.

#### 3.4.3 DHJSD is able to modulate ECM and inflammatory mediator secretion in LPS-treated NPCs by regulating macrophages

**Figure 15:**
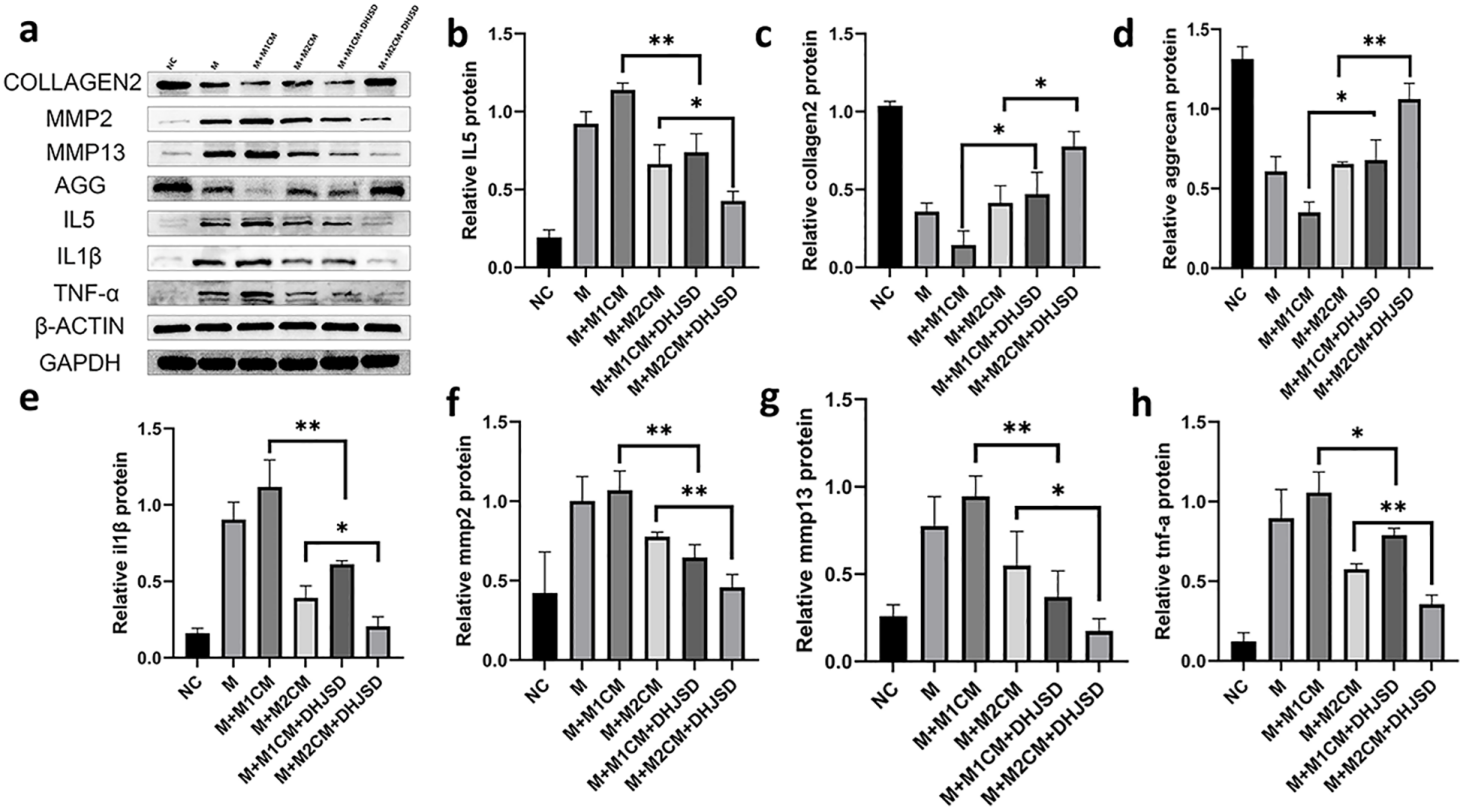
DHJSD modulates ECM and inflammatory mediator secretion in LPS-treated NPCs by regulating macrophage polarization. Representative Western blot bands of aggrecan, collagen 2, MMP13, MMP2, IL-5, IL-1β, and TNF-α protein expression in NPCs under different co-culture conditions **(a)**. Quantitative analysis of aggrecan, collagen 2, MMP13, MMP2, IL-5, IL-1β, and TNF-α protein expression levels **(b–h)**. Data are presented as mean ± SD (n = 3 independent experiments). Statistical comparisons were performed using one-way ANOVA followed by Tukey’s *post hoc* test. *p < 0.05, **p < 0.01.

To investigate the effect of DHJSD in modulating the ECM and inflammatory mediator secretion of LPS-treated NPCs by modulating macrophages, we cultured macrophages in medium containing DHJSD-drug-containing serum during polarization M1 and M2 before extracting M1CM and M2CM and co-culturing them with a model of NPCs. The expression of ECM aggrecan and collagen 2 was found to be better in the DHJSD treatment group than in the corresponding model group by immunoblotting, and the expression of MMP13, MMP2, and inflammatory factors IL-5, IL-1β, and TNF-α was lower than in the corresponding model group (**Figures 15a–h**). From this, we can conclude that DHJSD is able to modulate ECM and inflammatory mediator secretion in LPS-treated NPCs by regulating macrophages.

#### 3.4.4 DHJSD significantly inhibited M1 macrophage polarization and promoted M2 macrophage polarization

As can be seen by flow cytometry results, the addition of DHJSD resulted in a lower rate of positive M1 macrophage indication markers than in the no-DHJSD group and a higher rate of positive M2 macrophage surface markers than in the no-DHJSD group. Combined with the results of previous experiments, the following results can be concluded: DHJSD inhibits M1 macrophage polarization and promotes M2 macrophage polarization. Combined with the results of previous experiments, it can be concluded that DHJSD can inhibit M1 macrophage polarization and promote M2 macrophage polarization to modulate the immune-inflammatory response and thus ameliorate IVDD.

## 4 Discussion

Although the pathomechanisms of LBP are still not fully understood, it is anticipated that over 84% of the world’s population will suffer from LBP. IVDD is a significant pathogenetic component in LBP (30). Most current views suggest that the immune-inflammatory response is strongly associated with the variety of procedures that occur in IVDD (31). Specifically, macrophage-mediated immune-inflammatory responses are strongly associated with IVDD (10, 32–34). However, the exact mechanism of action of the macrophage-mediated immune-inflammatory response in IVDD is unknown. Therefore, we will investigate the mechanism of the role of immune-inflammation in IVDD in our research and offer guidance for the clinical treatment of IVDD. In Western countries, pharmacotherapy for IVDD is often limited to symptomatic relief with anti-inflammatory agents, which do not reverse the degenerative process. In contrast, traditional Chinese medicine compound prescriptions have the synergistic effect of multiple components and multiple targets. DHJSD is a traditional Chinese medicine formula that has been under long-term study by our group, and we have validated that this formula can ameliorate IVDD through a number of targets and pathways to ameliorate LBP in sufferers of IVDD. A systematic review of 31 randomized controlled trials (3915 patients) showed that DHJD alone or in combination improved clinical symptoms such as pain and straight leg raising angle in patients with lumbar disc herniation (35), and IVDD is considered to be the fundamental cause of the occurrence and development of lumbar disc herniation (36). And a meta-analysis of 14 randomized controlled trials (1560 patients) showed that modified DHJSD is superior to diclofenac sodium and other Western drugs in the total effective rate, cure rate, and visual analog scale improvement, and no obvious adverse reactions were reported (37). All the above results indicate that DHJSD is effective in the treatment of IVDD.

But the optimal therapeutic time node as well as its mechanism of action to modulate the immune-inflammatory response are not yet clear. In this research, we elucidated the mechanism of action of DHJSD-regulated immune inflammation in IVDD and explored the optimal therapeutic time point for DHJSD.

Firstly, we compared different cellular gene expression between IVDD sufferers and healthy persons using a single-cell sequencing approach and identified macrophage-related genes. Gene expression between IVDD sufferers and healthy persons was then analyzed by bioinformatics, and key differentially expressed genes were obtained. Then, 10 key genes associated with immune inflammation were further identified in combination with the PPI network, WGCNA analysis, etc. Of the 10 core genes identified by bioinformatics analysis, six (COL2A1, ACAN, MMP2, MMP13, TNF-α, and IL-1β) were experimentally confirmed at the protein level in our study. Consistent with the bioinformatics predictions, Western blot analysis showed that the disc degeneration model significantly reduced the expression of aggrecan and collagen 2, while increasing the expression of MMP2, MMP13, TNF-α, and IL-1β. Importantly, DHJSD treatment reversed these changes, further demonstrating the functional relevance of these core genes in the effect of DHJSD treatment. We finally clarified through *in vivo* and *in vitro* studies that DHJSD is able to modulate the immune inflammatory response to ameliorate IVDD by regulating macrophage polarization.

The current discussion of the pathophysiology of IVDD focuses on three main ideas, one of which is the immuno-inflammatory theory (38). In 1995, it was discovered that herniated discs can produce MMPs and inflammatory mediators (39). In 2002, it was clearly suggested that herniated disc tissue produces pro-inflammatory mediators. Macrophages are essential for the regulation of inflammatory mediators, MMPs, and specific cytokines in the disc (40). Starkweet found that salient NP tissue or disc fragments activate the neuroimmune system, inducing the release of large numbers of inflammatory cells, including macrophages (41). Wang also found that when the blood-NP barrier is compromised, NP triggers an immune response, with intervertebral disc cells and macrophages producing inflammatory factors and MMPs (42). It has been found that intervertebral discs can release monocyte chemotactic protein 1 (MCP-1) to amplify macrophage infiltration and inflammatory responses (43), and co-culture of degenerating NP cells with macrophages releases more positive factors that promote IVDD (44). From this, we can see that macrophage-mediated immune-inflammatory responses are involved in the pathogenesis of IVDD.

Interestingly, current research on IVDD is more biased toward cell death modalities, but what has been overlooked is that none of it is free from an immune-inflammatory response. Mass spectrometry analysis identified a complex array of bioactive components in DHJSD, as summarized in **Supplementary Table 2** (water extract) and 3 (ethanol extract). Among these, several compounds have been previously reported to possess immunomodulatory properties that may contribute to the observed effects on macrophage polarization. Notably, paeoniflorin, a monoterpene glycoside identified in both water and ethanol extracts, has been shown to inhibit M1 macrophage polarization and promote M2 polarization via suppression of the NF-κB signaling pathway (45). Glycyrrhizic acid, the major triterpene saponin from Glycyrrhiza uralensis, exhibits broad anti-inflammatory effects and has been reported to regulate macrophage phenotype switching (46). Quercetin and apigenin, flavonoids present in the ethanol extract, are known to suppress pro-inflammatory cytokine production and modulate macrophage polarization (47, 48). Additionally, liquiritin, epicatechin, and coumarin derivatives identified in DHJSD have demonstrated anti-inflammatory activities in various experimental models (49, 50).

While the present study did not isolate or test individual compounds, the presence of these bioactive constituents suggests that the immunomodulatory effects of DHJSD may result from the synergistic actions of multiple components rather than a single active ingredient. This is consistent with the traditional concept of Chinese herbal formulas, where therapeutic efficacy is often attributed to the combined effects of multiple constituents. Future studies employing activity-guided fractionation, network pharmacology, and targeted compound validation are warranted to identify the specific active components responsible for DHJSD’s regulation of macrophage polarization in IVDD. The formation of active caspase 1 in the classical pathway requires the activation of inflammatory vesicles, whereas the non-classical pathway requires more of an inflammatory response to lead to cell death in studies of cellular pyroptosis (51). Inflammatory factors are also involved in apoptosis (52, 53). It is easy to see how focusing on the immune-inflammatory response and associated immune cells may become an important strategy for treating IVDD. The importance of macrophages in immune inflammation is now recognized, but the mechanisms involved remain to be explored.

This study confirmed that DHJSD mainly regulates the immune inflammatory response by regulating macrophage M1/M2 polarization. On this basis, macrophages are the core cells of innate immunity, and their polarization can further affect the adaptive immune response through antigen presentation and cytokine secretion. The observation of T cells in single-cell sequencing in this study suggests that modulation of macrophage polarization during DHJSD treatment may have broader immunomodulatory effects. However, whether DHJSD affects adaptive immune responses by regulating macrophage polarization and the molecular mechanisms remain to be elucidated in future experiments.

## 5 Conclusion

In conclusion, we discovered that macrophages play a major role in the pathophysiology of IVDD and created a predictive model for the disease based on immuno-inflammatory hub genes such as aggrecan, collagen 2, MMP13, MMP2, IL-5, IL-1β, and TNF-α. Ultimately, we showed that DHJSD could reduce IVDD by preventing disc inflammatory reactions and the breakdown of ECM by boosting M2 macrophage polarization and suppressing M1 macrophage polarization. It is unclear which herbal component of DHJSD or which components regulate macrophages in IVDD by which specific pathway, despite recent advancements in the methods by which DHJSD affects macrophage polarization to relieve IVDD. We intend to further clarify the precise active elements in DHJSD that can successfully alleviate IVDD in order to progress the treatment of IVDD and even LBP. Going forward, we will continue to concentrate on the investigation of macrophage polarization in IVDD.

## Data Availability

The original contributions presented in the study are included in the article/**Supplementary Material**. Further inquiries can be directed to the corresponding authors.

## Supplementary material

Supplementary Table 1 The Chinese name, scientific name, genus, family, medicinal part, and batch number of the herbs in DHJSD.

Supplementary Table 2 The active pharmaceutical ingredients purified in water from DHJSD.

Supplementary Table 3 The active pharmaceutical ingredients purified in ethanol from DHJSD.
